# Postallogeneic stem cell transplant Hodgkin lymphoma: Rare presentation of an uncommon occurrence

**DOI:** 10.1002/ccr3.2095

**Published:** 2019-06-18

**Authors:** Raheel iftikhar, Qamar Un nisa Chaudhry, Tariq Mehmood Satti, Syed Kamran Mahmood, Tariq Ghafoor

**Affiliations:** ^1^ Armed forces Bone marrow transplant centre Rawalpindi Pakistan

**Keywords:** aplastic anemia, Epstein‐Barr Virus, Hodgkin lymphoma

## Abstract

Post‐transplant lymphoproliferative disorders are rare but potentially life‐threatening complication of HSCT. Although not frequently reported but PTLD can occur as a late post‐transplant complication in HSCT recipients. A high index of suspicion should be kept for early diagnosis of these disorders as delay in diagnosis can have catastrophic implications.

## 1 INTRODUCTION

Post‐transplant lymphoproliferative disorder (PTLD) is rarely reported in matched sibling donor (MSD) transplants of aplastic anemia (AA), and occurrence of Hodgkin lymphoma in this subgroup is extremely uncommon. Our patient, a 7‐year‐old girl, underwent MSD transplant for AA and developed EBV‐driven Hodgkin lymphoma after tapering of immunosuppression.

Post‐transplant lymphoproliferative disorders (PTLDs) represent heterogenous groups of clonal disorders occurring after solid organ and hematopoietic stem cell transplantation. Most of PTLDs are EBV driven and have a frequency of 3.2% in stem cell transplant recipients and 1.1% in matched sibling donor (MSD) transplants.^1^ Risk factors of PTLD include unrelated donor and haploidentical transplants, use of T‐cell depleting conditioning and higher recipient age. The underlying mechanism is failure of newly instituted donor immune system to control EBV‐infected host cells due to profound T‐cell immune suppression.

Epstein‐Barr Virus (EBV) DNA monitoring is done in these high‐risk patients to detect reactivation of EBV and institution of pre‐emptive measures like reduction of immunosuppression and treatment with rituximab. There are only few case reports of PTLD in patients of AA undergoing MSD transplant^2^ but to our knowledge this is first reported case of EBV‐driven Hodgkin lymphoma occurring after tapering of immunosuppression in patient undergoing MSD HSCT for AA.

## 2 CASE REPORT

A 7‐year‐old girl presented with 1‐week history of high‐grade fever and gum bleeding. There was no significant past medical history. She was born to consanguineous parents at full term by spontaneous vaginal delivery and was vaccinated as per immunization schedule. There was no family history of bleeding disorder, bone marrow failure, or hematological malignancy. On clinical examination, she had gum bleeding. There was no palpable lymphadenopathy or visceromegaly. Complete blood counts showed pancytopenia with hemoglobin of 6.2 g/dL, absolute neutrophil count (ANC) 0.3 × 10^9^/L, and platelet count of 8 × 10^9^/L. Bone marrow examination showed hypocellular marrow with 10 percent cellularity, absence of abnormal infiltrate or reticulin fibrosis, consistent with severe aplastic anemia. Cytogenetics was normal 46 XX, screening for Fanconi anemia, paroxysmal nocturnal hemoglobinuria, and secondary causes of aplastic anemia including viral serology, autoimmune profile, and thyroid profile were all negative. She underwent allogeneic stem cell transplant with her fully HLA matched brother. There was no ABO mismatch, and both donor and recipient were seropositive for cytomegalovirus (CMV) and Epstein‐Barr Virus (EBV). Conditioning regimen used was cyclophosphamide 200 mg/kg and Thymoglobulin 10 mg/kg, and cyclosporine was given for graft versus host disease (GVHD) prophylaxis. She achieved neutrophil engraftment on day + 14 and had uncomplicated early post‐transplant course except for febrile neutropenia. Her late post‐transplant course was also uneventful, and she had secure trilineage engraftment with complete donor chimerism and adequate B‐cell and T‐cell immune reconstitution by 1 year post‐transplant. Tapering of immunosuppression was started at 1 year and was stopped at 14 months post‐transplant.

Four weeks after stopping immunosuppression, she was seen in outpatient clinic with high‐grade fever and bilateral submandibular and cervical lymphadenopathy. Complete blood counts showed pancytopenia with WBC 0.95 × 10^9^/L, hemoglobin 9.7 g/dL, and platelets 43 × 10^9^/L. Contrast enhanced CT scan showed bilateral cervical, submandibular, mediastinal, and abdominal lymphadenopathy.

Bone marrow examination revealed markedly hypocellular marrow consistent with secondary graft rejection and PCR for short tandem repeats showed 50% donor Chimerism. Her PCR for cytomegalovirus (CMV), adenovirus, and human herpes virus‐6 (HHV‐6) were all negative. PCR for Epstein‐Barr virus (EBV) was positive (86 000 copies) demonstrating EBV reactivation. Excisional biopsy of cervical lymph node showed Hodgkin disease (Mixed cellularity type). Immunohistochemistry showed weak positive CD45, strong positivity for CD15, CD30, and PAX 5 in Reed‐Sternberg cells, and CD20 and EBV positivity in background cellular infiltrate. CD10 and BCL‐2 were negative. These findings were consistent with EBV‐associated post‐transplant lymphoproliferative disorder (HD stage III‐B) and secondary graft rejection. Treatment was started with Rituximab and COPDAC chemotherapy for Hodgkin lymphoma. She had an initial response which was documented by regression of lymph nodes but her pancytopenia persisted and repeat STRs after 4 weeks showed only 10 percent donor chimerism. She developed febrile neutropenia and multi‐organ dysfunction syndrome requiring broad‐spectrum parenteral antibiotics, amphotericin B, G‐CSF, granulocyte transfusions, intravenous immunoglobulin, and antiviral treatment. Her repeat PCR for EBV was negative 4 weeks later, and chemotherapy was continued. Despite aggressive management, her multi‐organ dysfunction worsened, and she could not be salvaged and expired.

## 3 DISCUSSION

Epstein‐Barr Virus belongs to human herpes‐4 (HHV4) family and is a double‐stranded DNA virus. Seropositivity of more than 90% is documented before adult life. It infects B cells via interaction with surface CD21 and gp350 glycoprotein and assumes latency after primary infection.^3^ Profound immunosuppression postallogeneic HSCT leads to activation of LMP1‐mediated proliferative signals via nuclear factor kappa B pathway. Moreover, BHFR1 exerts a BCL‐2 like activity and blocks apoptosis giving proliferative advantage and malignant transformation of EBV‐infected cells.^4^ PTLD post‐HSCT are almost exclusively EBV related and carries high mortality ranging from 35% to 80%.^5^ Haploidentical and unrelated HSCT, T‐cell depletion, GVHD, and high recipient age are well‐documented risk factors for PTLD in HSCT recipients. As per WHO 2017 classification, PTLD is categorized into six types: three types of nondestructive PTLD (plasmacytic hyperplasia, infectious mononucleosis‐like PTLD, and florid follicular hyperplasia), polymorphic PTLD, monomorphic PTLD, and classic Hodgkin lymphoma‐like PTLD. Most of PTLD in HSCT recipients develop 2‐4 months post‐transplant and only 4% occur after 12 months^6^ (as seen in our patient).

As per ECIL‐6 guidelines, risk for EBV‐PTLD can be classified as low risk (auto‐HSCT), standard risk (MSD allo‐HSCT without high risk factors, haplo‐PTCy HSCT), and high risk (MSD with at least one risk factor, MUD, alternative donors including CBT).^7^ ECIL recommends that EBV seropositivity testing should be done for both donor and recipient before HSCT. Post‐transplant serial monitoring with EBV DNA PCR needs to be done, starting at 4 weeks post‐transplant and continuing weekly till 4 months post‐HSCT. Longer monitoring may be needed for patients with active GVHD, haploidentical HSCT and for patients with poor T‐cell reconstitution post‐transplant. In many institutional guidelines, viral load of more than 10 000 copies/mL is considered for pre‐emptive treatment with rituximab and tapering of immunosuppression.^8^

Therapeutic approaches for EBV‐PTLD includes administration of Rituximab, reduction of immunosuppression, donor lymphocyte infusions, EBV‐specific cytotoxic T‐lymphocytes (CTL) and chemotherapy for specific PTLD. As per ECIL guidelines, patients with standard risk for PTLD do not require pre‐emptive EBV DNA PCR monitoring, and our patient fell in standard risk category, but developed fatal PTLD highlighting variable presentation of PTLD and a need to reconsider high‐risk criteria for PTLD.

Our case is unusual in number of aspects; it is one of few cases of PTLD in AA patients; it developed late post‐transplant, occurred after tapering of immunosuppression, and associated with secondary graft rejection; and histological type was Hodgkin lymphoma, which is least common type of PTLD. EBV reactivation was most likely responsible for malignant transformation of B cells and secondary graft failure as evident by clinical signs and symptoms and high titer EBV viral load.

## CONFLICT OF INTEREST

None declared.

## AUTHOR CONTRIBUTION

RI: collected data, involved in medical writing, and reference citation. QU nC and TG: collected data and involved in medical writing. TMS and SKM: contributed to reference citation and collected data.
